# Zoonotic Parasites in Marmosets (*Callithrix* Spp.) From Southern Brazil: Insights From a One Health Perspective

**DOI:** 10.1111/jmp.70030

**Published:** 2025-08-17

**Authors:** Maysa Emanuely dos Santos, Matheus Azambuja, Denilton Vidolin, Rosimeire Nunes de Oliveira

**Affiliations:** ^1^ Department of General Biology Universidade Estadual de Ponta Grossa Ponta Grossa Paraná Brazil; ^2^ Departament of Structural and Molecular Biology and Genetics Universidade Estadual de Ponta Grossa Ponta Grossa Paraná Brazil

**Keywords:** *Callithrix*, enteroparasites, health surveillance, one health

## Abstract

**Background:**

Currently, six species of *Callithrix* are frequently observed in anthropized and degraded environments in different regions of Brazil. This occupation favors interactions with humans and increases the risk of infection by parasites with zoonotic potential. This study assessed the occurrence of enteroparasites in free‐ranging *Callithrix* spp. from different municipalities in Paraná state, Brazil, from a One Health approach.

**Methods:**

Faecal samples of 37 specimens of *C. penicillata* , *C. jacchus* , and hybrid species were analyzed.

**Results:**

The most frequent parasite forms observed included helminth eggs (Ascaridida, Rhabditida, Trichostrongylidae), and protozoan oocysts and cysts (Coccidia, Diplomonadida). Clinical evaluations of the animals indicated musculoskeletal abnormalities, dental changes, skin injuries, gastrointestinal signs, and low body weight.

**Conclusions:**

The presence of parasites with zoonotic potential highlights the need for ongoing surveillance of marmoset populations, underscoring the importance of maintaining a balanced relationship among the environment, humans, and animals within the One Health approach.

## 1 Introduction

Primates of the *Callithrix* genus, commonly known as marmosets, are small to medium‐sized neotropical monkeys belonging to the Callitrichidae family. This genus includes six recognized species: *C. jacchus* , *C. penicillata* , *C. kuhlii* , *C. geoffroyi* , *C. aurita* , and *C. flaviceps. Callithrix* species were distinguished from their closely related species by their diverse pelage coloration, ringed tails, and marked adaptability to various ecological niches [1]. These traits help explain their extensive distribution throughout the Brazilian Cerrado, Caatinga, Atlantic Forest biomes, and disturbed environments in urban areas [2]. Furthermore, according to Mittermeier (2009), some species adapt easily to anthropogenic environments [3].

Due to omnivorous behavior and high plasticity, namely the adaptive capacity of the *Callithrix* genus to explore different environmental niches, marmosets are frequently reported in urban and peri‐urban areas, particularly in the Northeast, Southeast, and Central‐West Brazilian geographical regions, with expanding records in the South [4]. Notably, *C. penicillata* , *C. jacchus* , and *C. geoffroyi* have been identified in several municipalities of Paraná state and coastal regions of Santa Catarina state, with occasional registers in Rio Grande do Sul state [5].

Habitat fragmentation, urban expansion, and environmental degradation have facilitated the dispersal of *Callithrix* species beyond their original ranges. This spatial expansion increases their exposure to anthropogenic pressures and elevates the risk of contact with humans and domestic animals, contributing to zoonotic disease transmission [1, 2, 3, 4, 5, 6]. Furthermore, the illegal wildlife trade exacerbates this issue by promoting the capture and transportation of these primates under unsanitary and stressful conditions, favoring the spread of infectious diseases, including parasitic infections [7].

In a One Health context, through the interdisciplinary approach between human, animal, and environmental health, the role of *Callithrix* spp. as reservoir animals of zoonotic pathogens is significant [8]. Previous studies have identified natural infections by *Plasmodium* spp., *Trypanosoma* spp., and *Leishmania* spp. in *C. jacchus* , as well as enteric parasites such as *Giardia duodenalis*, *Entamoeba coli* , and *Strongyloides* spp. in free‐ranging marmosets [9, 10, 11]. In addition, gastrointestinal parasites, including *Balantioides coli*, *Strongyloides* spp., *Trichuris* spp., oxyurids, *Hymenolepis nana*, and acanthocephala are also diagnosed in non‐human primates, often with implications ranging from asymptomatic infections to diarrhea, prostration, dyspnea, appetite loss, and severe dehydration [12, 13, 14, 15]. Infections caused by parasites that can determine zoonotic transmission cycles of public health concern increase the need for surveillance, management, and conservation of wild animals, under human supervision [16, 17].

Despite growing concern regarding wildlife parasitology, data on natural parasitism in *Callithrix* spp., particularly in southern Brazil, remain scarce. Such information is essential for guiding conservation strategies and understanding parasite–host relations in disturbed landscapes. Thus, this study aimed to evaluate the occurrence of enteroparasites in free‐ranging *Callithrix* spp. from different municipalities in Paraná state, southern Brazil, and correlate parasitic infections with clinical health parameters of the hosts. The findings are interpreted through a One Health view, highlighting the implications for zoonotic disease surveillance.

## 2 Materials and Methods

### 2.1 Origin and Capture of *Callithrix* Spp.

**FIGURE 1:**
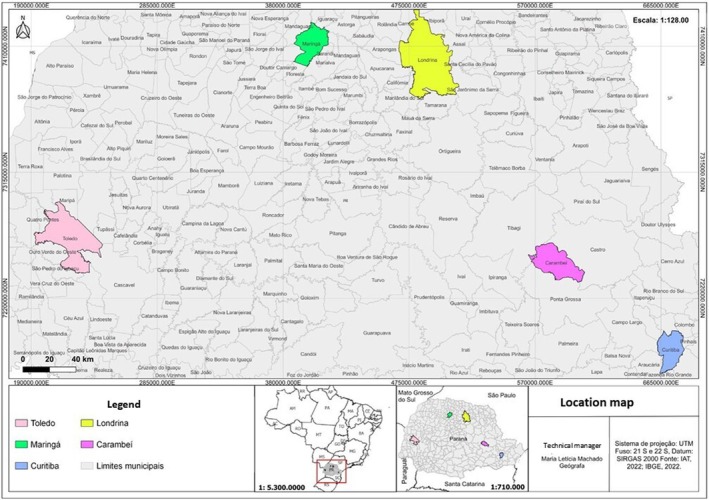
Location of the municipalities where the *Callithrix* spp. specimens were collected in the state of Paraná, southern Brazil. *Source:* Instituto Água e Terra (IAT), 2022; Instituto Brasileiro de Geografia e Estatística (IBGE), 2022. Software: Quantum GIS (QGIS).

The marmosets (*Callithrix* spp.) included in this study were captured in urban areas from Toledo, Curitiba, Londrina, Carambeí, and Maringá municipalities in Paraná state, Brazil's Southern region (Figure 1) by the Instituto Água e Terra (IAT), the environmental agency of Paraná State, or were derived from the voluntary delivery of animals to the IAT agency. Specimens were transported to the facilities of the Laboratory of Zoology at Universidade Estadual de Ponta Grossa (UEPG). A total of *n* = 37 animals were evaluated between March 2023 and September 2024. Specimens were identified following morphological characteristics according to Rylands and collaborators [1].

### 2.2 Clinical Evaluation and Faecal Sample Collection

Following anaesthesia, the animals underwent clinical evaluation by a veterinarian. The examination included assessment of mucous membranes, body weight, dental condition, spinal alignment, and nutritional status. Faecal samples were collected directly from the rectal ampulla or the interior of the transport cage and subsequently transferred to plastic containers containing 5% formalin solution for preservation until laboratory analysis.

### 2.3 Coproparasitological Analysis

Two standard parasitological concentration techniques were employed: spontaneous sedimentation according to Hoffman, Pons and Janer (1934) [18] and centrifuge‐sedimentation according to Ritchie (1948) [19], which is the most adequate for helminths and protozoa diagnosis. Microscopic analyses were performed using a light microscope at 10× and 40× magnifications. At least three slides were prepared and examined for each method. All parasitic forms found were photographed for identification.

### 2.4 Ethics Statement

All procedures were in accordance with the Ethics Committee for the Use of Animals (CEUA‐UEPG), under protocol nº. 8503631, and were approved by the Biodiversity Authorization and Information System (SISBIO), under protocol nº. 22000075069‐4.

## 3 Results

**FIGURE 2:**
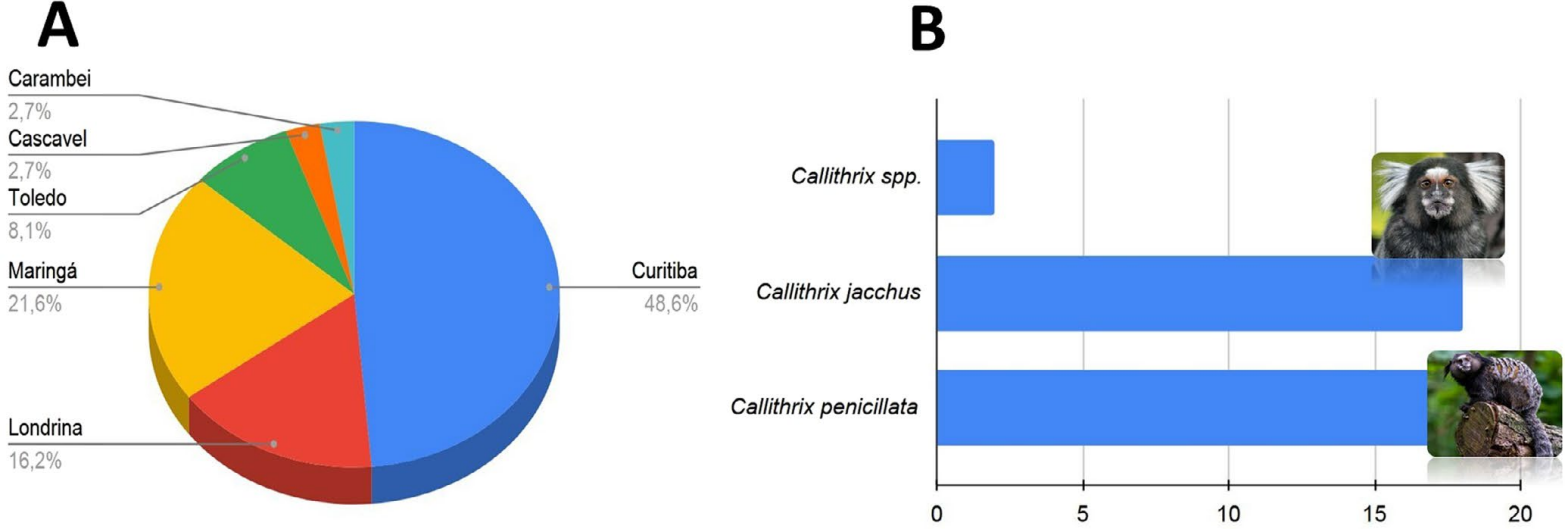
Percentage of *Callithrix* spp. specimens studied according to municipality of origin in the Paraná state (A), and prevalence of intestinal parasitism (B). *C. jacchus‐*Image—Julian, SB.; *C. penicillata*‐Image‐Ouwesok. *Source:* (https://museucerrado.com.br).

Faecal samples from 37 specimens of *Callithrix* spp. collected in different municipalities of Paraná, southern Brazil, were analyzed. Most individuals originated from Curitiba (18), Maringá (8), and Londrina (6) (Figure 2A). Coproparasitological analyses were performed on all specimens to assess the presence of enteric parasites. The overall positivity rate was 24.3%, with *C. penicillata* showing the highest prevalence of infection (Figure 2B).

**FIGURE 3:**
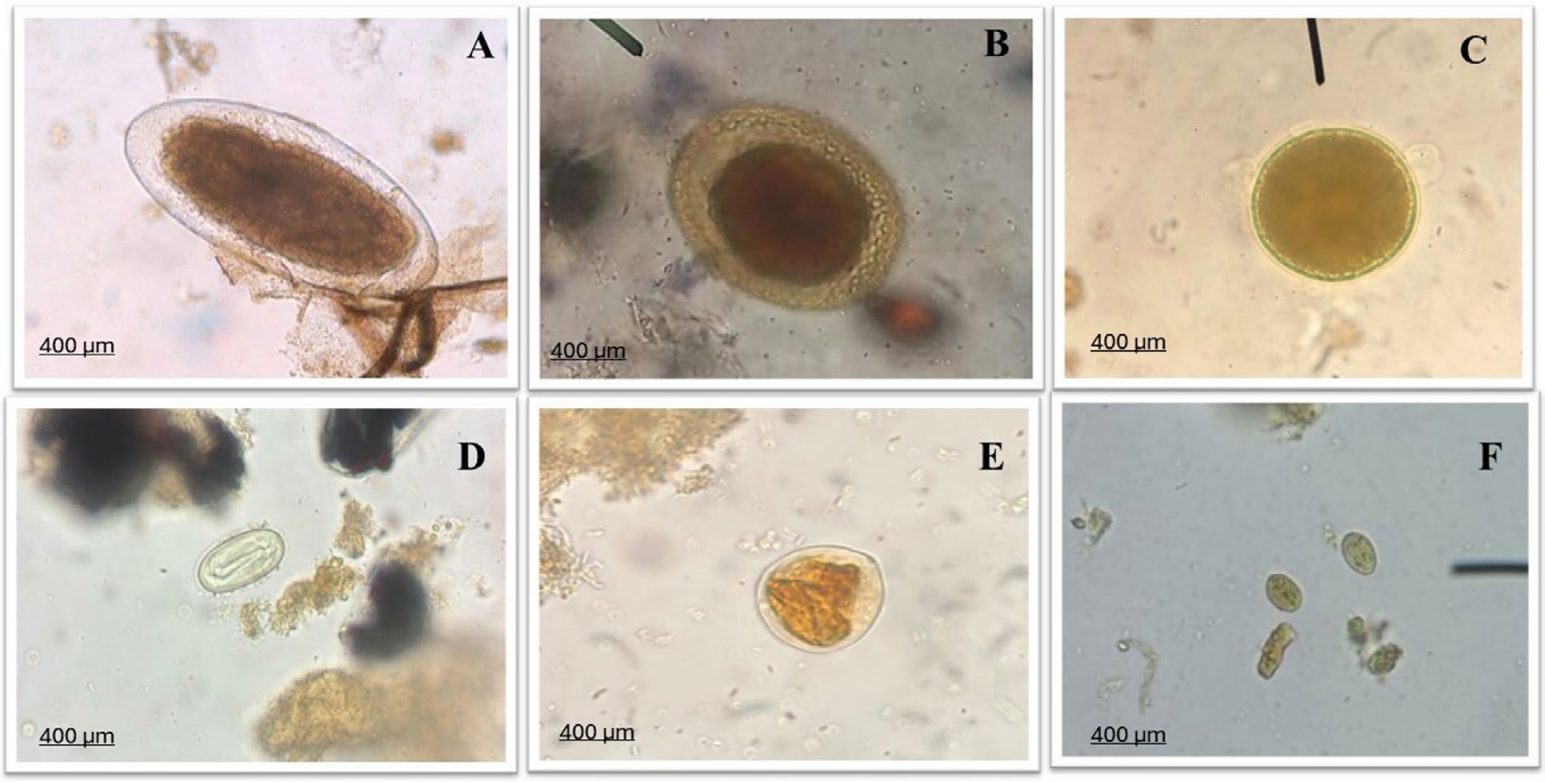
Parasitic forms observed in faecal samples of *Callithrix* spp. captured in different municipalities of Paraná, Brazil. (A) *Trichostrongylidae* egg (400×); (B, C) *Ascaridida* eggs (400×); (D) *Rhabditida* egg (400×); (E) *Coccidia* oocyst (400×); (F) *Giardia* spp. cyst (*Diplomonadida*) (400×).

Regarding the parasitic forms identified in the faecal samples, helminth eggs were most frequently observed, including representatives of the orders Ascaridida, Rhabditida, and Trichostrongylidae family (Figure 3). In addition, protozoan oocysts and cysts were detected, belonging to the orders *Coccidia* and *Diplomonadida* (Figure 3). However, species‐level identification of the parasites was not possible due to limitations in sample collection; the absence of adult forms; and the lack of molecular diagnostic analyses.

**FIGURE 4:**
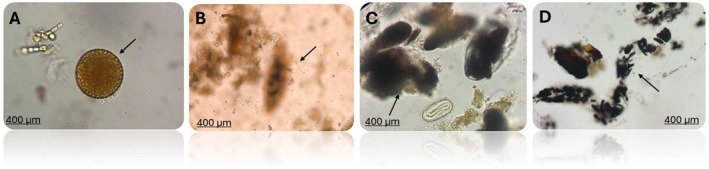
Photomicrographs of faecal artefacts (arrows) identified in *Callithrix* spp. captured in different municipalities of Paraná, Brazil (40× objective). (A) Pollen grain; (B) Vegetable cell; (C) Vegetable cells and starch granules; (D) Starch granule.

Additionally, a substantial quantity of faecal artefacts was observed during microscopic analysis (Figure 4). These findings are consistent with the omnivorous diet and foraging behaviour characteristic of *Callithrix* spp., providing relevant information about the dietary ecology of these primates. The presence of such artefacts may also influence parasite acquisition and the overall dynamics of intestinal parasitism in wild populations.

**FIGURE 5:**
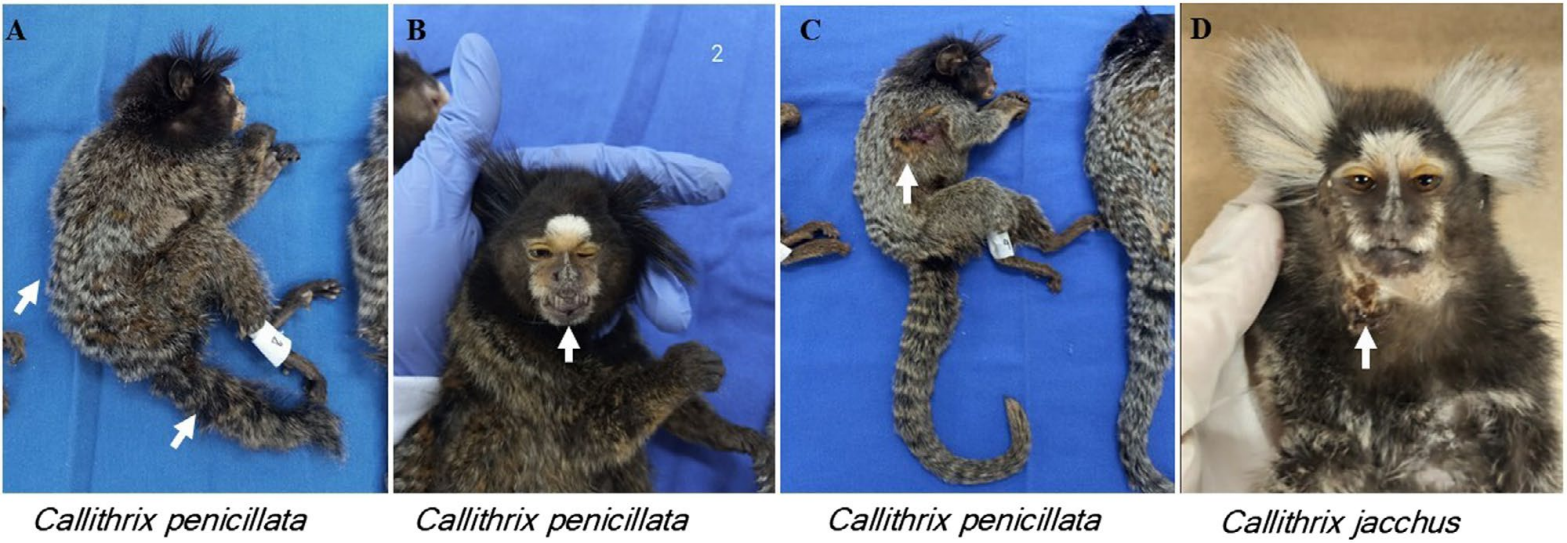
Health status of the animals evaluated in this study. (A) *Callithrix penicillata* showing vertebral changes—tail and spine; (B) *C. penicillata* presenting dental changes; (C) *C. penicillata* showing damage injuries to skin; (D) showing damage injuries to skin and alopecia.

The clinical condition of the marmoset was verified to correlate the implications of parasitism with animal and human health (Tables 1 and 2). Overall, the health condition of *Callithrix* spp. varied considerably. Some animals exhibited musculoskeletal abnormalities, including spinal, limb, amputated tail, dental changes, and skin injuries, while others showed signs of gastrointestinal distress and weight loss (Figure 5). A subset of specimens appeared clinically healthy and had an average body weight of 281 g. Clinical conditions of the marmosets were assessed to explore possible associations between parasitism and animal health. However, it is important to highlight that these clinical findings may result from factors unrelated to parasitic infection, since three specimens were clinically healthy despite parasitism, as detailed in Tables 1 and 2.

**TABLE 1:** General health parameters of *Callithrix* spp. specimens with positive results after coproparasitological analysis.

Species	Sex	Weight (kg)	Health status	Parasitism	Origin
*Callithrix penicillata*	Male	0.266	Healthy	Positive Technique: Hoffman and Ritchie	Curitiba, Paraná, Brasil
*Callithrix penicillata*	Male	0.226	Changes to the spine, half of the tail amputated	Positive Technique: Hoffman and Ritchie	Curitiba, Paraná, Brasil
*Callithrix penicillata*	Male	0.384	Healthy	Positive Technique: Hoffman and Ritchie	Curitiba, Paraná, Brasil
*Callithrix penicillata*	Female	0.335	Diarrheal, foul‐smelling, yellowish feces.	Positive Technique: Hoffman and Ritchie	Curitiba, Paraná, Brasil
*Callithrix jacchus*	Male	0.219	Healthy	Positive Technique: Hoffman and Ritchie	Londrina, Paraná, Brasil
*Callithrix jacchus*	Male	0.238	Amputated limb and a fractured tail	Positive Technique: Hoffman and Ritchie	Maringá, Paraná, Brasil
*Callithrix jacchus*	Female	0.303	Signs of neurological alterations, and trauma	Positive Technique: Hoffman and Ritchie	Maringá, Paraná, Brasil

*Note:* All fecal samples were analyzed using the Hoffman and Ritchie methods. The total content of the fecal sample from each specimen was analyzed by optical microscopy.

**TABLE 2:** Taxonomic diversity of enteroparasites identified in *Callithrix* spp. examined in this study and potential implications for human and animal health.

Taxonomic classification	Zoonotic species	Risk to human health	Health implications in *Callithrix* spp.	Transmission routes	Life stage detected	Zoonotic potential	References
Trichostrongylidae (Nematodes)	*Trichostrongylus* spp.	Enteritis, anemia, obstructions of organs such as bronchi and bronchioles	Mild GI disturbances, anemia, obstructions of organs	Ingestion of filarial larvae	Eggs	Low	[20, 21]
Ascaridida (Nematodes)	*Toxocara* spp.	Potential cause of larva migrans Lung, eye, tissue, nerve, and enteritis implications	Nutritional deficits, intestinal distress	Ingestion of embryonated eggs	Eggs and adults' worms	High	[22]
Rhabditida (Nematodes)	*Strongyloides* spp.	Gastrointestinal symptoms; Possible opportunistic infections	Gastrointestinal and pulmonary symptoms	Active penetration of filarial larvae; Ingestion of filarial larvae.	Rhabditoid and Filarial larvae	Moderate	[22, 23, 24]
Coccidia (Protozoa)	*Emeiria* ssp.	Enteritis, diarrhea, malabsorption Weight loss	Diarrhea, malabsorption	Ingestion of sporulated oocysts	Oocysts	Low to moderate	[25]
Diplomonadida (Protozoa)	*Giardia* spp.	Diarrhea, malabsorption Weight loss	Diarrhea, malabsorption Weight loss	Ingestion of cysts	Cysts	High	[26]

*Note:* Zoonotic potential based on literature reports and known transmission routes. Presence of these parasites in free‐ranging primates may indicate environmental contamination and contribute to One Health surveillance strategies.

## 4 Discussion

Environmental alterations, such as habitat fragmentation and increased proximity to anthropogenized environments, significantly influence the dynamics of transmission of pathogens, facilitating the spillover of diseases between wildlife, domestic animals, and humans [27]. This scenario is likely in large parts of the Brazilian Atlantic Forest, which has lost over 80% of its original vegetation cover and is a recognized biodiversity hotspot where environmental degradation may exacerbate the transmission of zoonotic diseases [3]. In the present study, most *Callithrix* spp. specimens were captured in the municipalities of Curitiba, Maringá, and Londrina, suggesting a predominance of individuals from urban environments. This distribution supports previous findings highlighting the adaptability of these primates to anthropogenically altered and fragmented habitats [28].

Parasitological analysis revealed an overall infection rate of 24.3%, with *C. penicillata* showing the highest frequency of helminth infections. The most common parasitic taxa identified were from the orders *Ascaridida* and *Rhabditida*, and the family *Trichostrongylidae*. The prevalence of helminth infections in *Callithrix* spp. may be closely related to their dietary habits. These omnivorous primates commonly feed on invertebrates, including insects that serve as intermediate hosts for various parasitic species. Members of the order *Coleoptera*, for example, represent a frequent dietary component for *Callithrix* spp. and may facilitate the ingestion and transmission of helminth parasites [29, 30, 31]. In addition to helminths, evolutionary stages of protozoa, namely *Coccidia* and *Diplomonadida*, were also identified in the faecal samples. These findings are consistent with previous studies reporting enteric protozoa in other *Callithrix* species, including genera such as *Entamoeba*, *Giardia*, *Cryptosporidium*, *Cystoisospora*, and *Balantidium* [31, 32, 33].

Research on protozoan parasites in *Callithrix* spp. in Brazil has seen a significant increase between 2003 and 2022 [12]. In contrast, helminthological investigations in these primates date back to the early 20th century, with records from as early as 1910. This chronological gap reflects the historical emphasis of parasitology on helminth infections and the more recent expansion of studies into protozoan diversity, which has significantly broadened our understanding of the parasitic landscape affecting neotropical primates [12, 13, 14, 15, 16, 17, 18, 19, 27, 28, 29, 30, 31, 32, 33, 34, 35].

A review by Solórzano‐García and Pérez‐Ponce de León, 2018 [36], identified eight protozoan species in wild New World primates: *Giardia intestinalis*, *Isospora arctopitheci*, *Leishmania infantum*, *Plasmodium brasilianum*, *P. malariae*, *Toxoplasma gondii*, *Trypanosoma cruzi*, and *T. minasense*. Many of these organisms have recognized zoonotic potential, emphasizing the need for surveillance programs to better assess the impact of protozoan infections on host health and the associated risks in areas of close human–primate interaction.

The detection of *Giardia duodenalis* in faecal samples of *Callithrix* spp. represents a significant finding for animal and public health due to its recognized zoonotic potential. Genetic similarities between *G. duodenalis* assemblages A and B commonly found in humans, domestic animals, and non‐human primates highlight the risk of cross‐species transmission [26, 37]. Transmission occurs through ingestion of water or food contaminated with cysts and may lead to gastrointestinal symptoms such as diarrhea, dehydration, nutrient malabsorption, and enteritis.

In *Callithrix* spp., particularly in habitats with limited access to clean water or adequate nutrition, *Giardia* infections can severely impact overall health, welfare, and survival, emphasizing the need for parasitological surveillance and sanitary management in human–primate interface areas. *G. duodenalis* infections have also been reported in *Callithrix argentata* , *Cercopithecus erythrogaster* , *Macaca fuscata* , *M. mulatta* , and *Alouatta pigra* , reinforcing its widespread occurrence across multiple primate taxa [21, 38].

Similarly, the presence of helminths from the family *Trichostrongylidae* in *Callithrix* spp. faeces is worrying. These gastrointestinal nematodes are typically associated with ruminants, but can also infect primates, particularly in captive or peri‐urban environments, where natural ecological barriers are diminished [21]. Infections may manifest as diarrhoea, weight loss, and anemia symptoms that compromise host health and welfare, especially in primates like *Callithrix jacchus* (common marmoset). Although *Trichostrongylidae* are more prevalent in herbivorous hosts, their detection in omnivorous primates suggests possible cross‐transmission, especially in shared habitats or mixed‐species environments [12].

Coprological analyses in this study also revealed the presence of helminth eggs from the order *Ascaridida* in *Callithrix jacchus* . Infections by *Ascaridida* can negatively impact the health of marmosets, especially in juvenile, malnourished, or immunocompromised individuals. These enteroparasites compete with the host for essential nutrients, potentially leading to diarrhoea, weight loss, and a generally debilitated state, which may impair survival and reproductive success. Moreover, *Ascaridida* infections in non‐human primates pose a potential zoonotic threat. Species of the genus *Toxocara*, for example, are well‐documented human pathogens that can cause toxocariasis, a condition associated with visceral and ocular complications [22].

The detection of nematodes from the order *Rhabditida* in the faeces of *Callithrix* spp. also raises important concerns for animal and human health, as some members of this group hold zoonotic significance. Notably, nematodes of the genus *Strongyloides* are recognised for causing a spectrum of intestinal infections, ranging from asymptomatic to severe cases. Clinical manifestations can include chronic diarrhoea, weight loss, and, in immunocompromised individuals, systemic dissemination [23, 39]. In *Callithrix* spp., parasitism by *Strongyloides* spp. is particularly concerning due to the zoonotic potential of species such as *Strongyloides stercoralis*. Human infection may occur in shared environments, such as urban parks and peri‐domestic areas where marmosets and humans coexist [24]. In humans, *S. stercoralis* is capable of causing internal autoinfection, potentially leading to hyperinfection syndrome and disseminated strongyloidiasis. This severe condition can affect multiple organs and is associated with high mortality rates, reaching up to 87% in untreated cases [40].

The feeding behaviour of *Callithrix* spp. is also pointed out as an important factor in understanding the dynamics of parasitic infections. The presence of food artefacts in faecal samples, such as pollen grains and vegetable cells, aligns with a diverse diet typical of *Callithrix* species. Therefore, recognize that the artefacts observed in this study are common in omnivorous species. In addition, their detection does not allow us to establish a relationship with the parasitism observed in the animals studied. This feeding pattern has been previously described by Rylands and Faria (1993) [41] and Michaud and collaborators [39], who reported that marmosets are primarily exudative‐insectivorous but also frugivorous. With the increasing destruction and fragmentation of natural habitats, *Callithrix* spp. have frequently moved closer to anthropogenized environments. This proximity facilitates sharing food sources, such as fruit and food scraps intentionally provided or accessed in household waste, which can promote the acquisition of parasites and compromise the health of these animals [42].

A study conducted by Tavela and collaborators [42] in Viçosa, Minas Gerais, southeastern Brazil (20°45′S and 42° 52′W) demonstrated that wild hybrid marmosets—*Callithrix* spp.—living in an environment with high human activity are exposed to intermediate hosts of helminths, such as urban insects (cockroaches), associated with parasites such as Ancylostomatidae, Strongylidae, *Prosthenorchis* sp., and Dilepididae species.

The dietary plasticity of *Callithrix* species is associated with the high ecological adaptability of this group. As reported by Rylands (1996) [41, 43], during periods of fruit scarcity, marmosets may increase their reliance on plant gums, allowing them to maintain a nutritionally adequate diet and sustain reproductive success, contributing to more stable and healthier social groups.

Although habitat fragmentation should be considered not only as a factor that may increase exposure to parasites but also as a driver of dietary shifts that could mitigate parasitic risks, in the present study, some specimens arrived at the institution in a visibly healthy condition and with low parasitic burden.

However, we acknowledge that this study has some limitations that should be considered when interpreting the results: (i) the small number of specimens evaluated (*n* = 37) limits the possibility of establishing more robust correlations; (ii) the absence of molecular analyses made it impossible to accurately identify the parasitic species at the genetic level, restricting the taxonomic classification to the genus or family of the agents detected; and (iii) no studies have been conducted on the quality of the diet of *Callithrix* spp., making it impossible to establish accurate associations between eating habits, occurrence of parasitism, and clinical symptoms.

## 5 Conclusion

This study underscores the importance of investigating the parasitological fauna of *Callithrix* species, correlating parasite presence with host health status and habitat characteristics. Such an approach enables a broader understanding of the factors influencing parasitism and its impact on animal health, within a One Health framework offering essential insights for conservation and public health strategies in areas of human–nonhuman primate interface.

## Conflicts of Interest

The authors declare no conflicts of interest.

## Data Availability

The data that support the findings of this study are available from the corresponding author upon reasonable request.
